# The Psychosocial Impact of COVID-19 on Older Adults with Cancer: A Rapid Review

**DOI:** 10.3390/curroncol29020053

**Published:** 2022-01-28

**Authors:** Ridhi Verma, Heather M. Kilgour, Kristen R. Haase

**Affiliations:** 1 School of Healthcare Sciences, Cardiff University, Cardiff CF10 3AT, UK; ridhiverma.in@gmail.com; 2 School of Nursing, The University of British Columbia, Vancouver Campus, T201-2211 Wesbrook Mall, Vancouver, BC V6T 2B5, Canada; heather.kilgour@ubc.ca

**Keywords:** cancer, older adults, mixed methods, COVID-19, quality of life, patient experience, qualitative methods

## Abstract

Background: Older adults with cancer are amongst the most vulnerable population to be negatively impacted by COVID-19 due to their likelihood of comorbidities and compromised immune status. Considering the longevity of the pandemic, understanding the subjective perceptions and psychosocial concerns of this population may help ameliorate the psychological aftermath. In this review, we systematically analyze the literature surrounding the psychosocial impact and coping strategies among older adults with cancer within the context of COVID-19. Methods: We conducted a rapid review of literature following PRISMA guidelines between January 2020 to August 2021 using (1) MEDLINE, (2) Embase, (3) CINAHL, and (4) PsychINFO and keyword searches for “cancer” and “COVID-19” focused on adults 65 years or older. Results: Of the 6597 articles screened, 10 met the inclusion criteria. Based on the included articles, the psychosocial impact of COVID-19 was reported under four domains, (1) impact of COVID-19 on quality of life (QoL), (2) concerns related to COVID-19, (3) coping with the impact of COVID-19, and (4) recommendations for future care. Results pertaining to perceived quality of life were inconsistent across the included articles. The most common concerns related to: contracting COVID-19, survivorship transitions, and feelings of isolation. Coping strategies reported by older adults included: spiritual care, lived experience, acceptance, and positive reinterpretation. Conclusions: We found many psychosocial impacts of the pandemic on older adults with cancer. The findings from this review can inform interventions related to shared decision-making and tailored patient care in the future.

## 1. Introduction

Nearly two years into the COVID-19 pandemic, it continues to impact individuals’ lives and health systems globally. This is particularly true of older adults with cancer, who may be among the most vulnerable to the effects of COVID-19 due to their immunocompromised status and increased presence of comorbidities [1,2,3,4,5]. Older adults diagnosed with both cancer and COVID-19 face increased mortality, hospitalization, and ICU admission [3,4,5]. In addition, the COVID-19 pandemic has caused significant disruption to the cancer care system, including a shift to virtual appointments, alternative treatment offerings, postponement of surveillance scans and surgeries, and diminished supportive care and survivorship services [6,7]. Older adult cancer survivors have described barriers to cancer treatment across the pandemic, including restricted attendance of caregivers at appointments, diminished access to healthcare services, and feeling less connected to their healthcare team [8,9]. Understanding the impact of these changes on the experiences of older adults with cancer is essential to learning how best to support older adults during this time.

Public health measures meant to diminish the spread of COVID-19, such as social distancing and stay-at-home mandates, have resulted in older adults spending more time alone, which may enhance feelings of isolation and loneliness. Recent reviews suggest that COVID-19 has created considerable anxiety, irritability, feelings of paranoia, and depression amongst community-dwelling older adults and people with serious comorbidities [10]. One Canadian study exploring loneliness amongst community-dwelling older adults found that 43.1% of older adults had experienced loneliness at least some of the time in the preceding week [11]. Factors associated with increased loneliness included having fair or poor health, changes to daily routine, and having a high concern for the pandemic [11]. These factors may be experienced differently by older adults with cancer, potentially exacerbating the impact of the COVID-19 pandemic on this patient population. With no clear ending of the COVID-19 pandemic in sight, understanding the psychosocial impact of the COVID-19 pandemic on older adults with cancer is crucial to provide patient- and family-centered care to this patient population.

In the face of the COVID-19 pandemic, older adults with cancer face intersecting vulnerabilities related to the physical effects of the virus, cancer-system changes, and the potentially isolating impacts of public health measures. The culmination of these effects on older adults warrants exploration as clinicians look to support older adults with cancer and prepare for future health crises. The purpose of this review is to synthesize the literature surrounding the psychosocial impact and coping strategies of older adults with cancer during the COVID-19 pandemic. Understanding how the pandemic has impacted older adults with cancer will help clinicians support them during subsequent waves of this pandemic, as well as plan for future pandemics.

## 2. Methods

To conduct this rapid review, we followed systematic review methodology and the updated preferred reporting items for systematic reviews and meta-analysis statement [12]. In alignment with best evidence on rapid review methods [13,14], we have streamlined the review process to rapidly produce information that is accessible to clinicians and decision-makers by limiting to publications in English only, limiting the search time frame, and having one reviewer for abstracts and data abstraction with a second reviewer ensuring abstraction is completed in a timely manner. 

### 2.1. Inclusion Criteria

- Peer-reviewed primary research of either qualitative, quantitative, or mixed-methods design.
- Published in the English language.
- Published since 2020 (which coincides with the start of the pandemic).
- Focused solely on older adults or those with a mean/median age of ≥65.

### 2.2. Exclusion Criteria

- Expert opinions, editorials, case reports/case studies, gray literature, and secondary research.

### 2.3. Information Sources and Search

We conducted a comprehensive search strategy for this rapid review through consultation with an experienced health sciences librarian. The electronic databases searched included: (1) MEDLINE, (2) Embase (3) CINAHL, (4) PsychINFO. We did not search the gray literature as we felt it would not add additional value. The results were imported into Covidence [15] systematic review software to facilitate the screening of abstracts and full text. The detailed search conducted in MEDLINE is outlined in the Supplemental Material (Listing S1).

### 2.4. Study Selection

**Figure 1:**
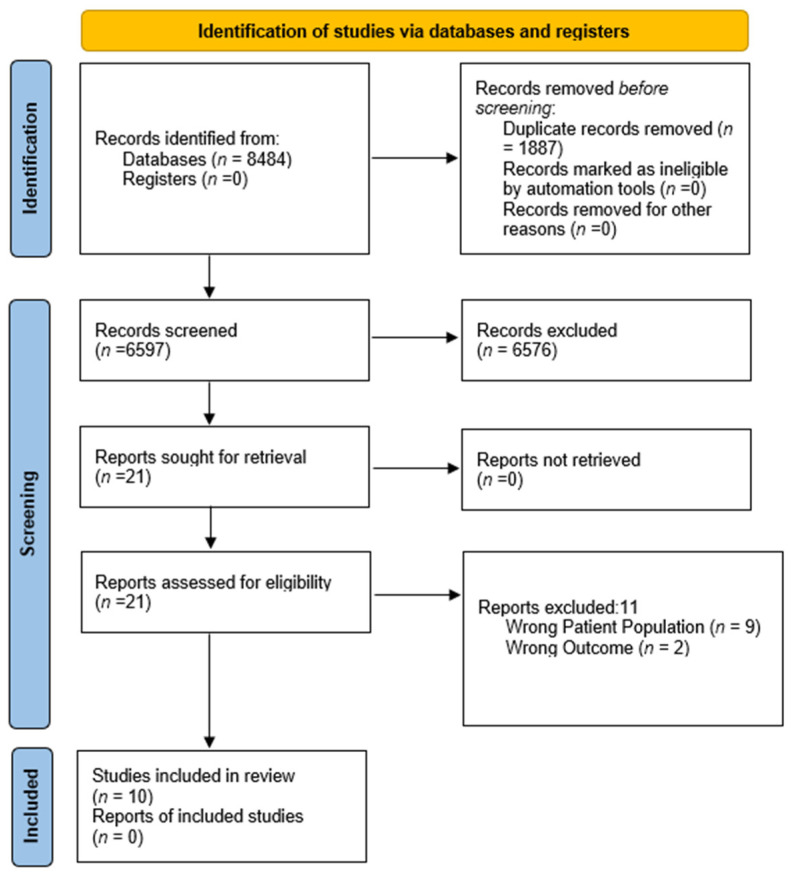
PRISMA flow diagram.

Since title and abstract screening were performed by one reviewer, we conducted training before screening, developed detailed inclusion/exclusion criteria, and held regular meetings to discuss any ongoing concerns. The review of the full texts was also conducted by one reviewer and verified by a second reviewer. The senior author (KH) resolved any conflicts. Figure 1 represents the PRISMA flow diagram outlining the study selection.

### 2.5. Data Abstraction

Data were collected using a standardized form in MS Excel, which included study characteristics (e.g., author, country, year, duration of data collection, and design), patient characteristics (e.g., target population and sample size, cancer diagnosis), aims of the study, outcomes along with corresponding outcome measures, and the study findings. 

### 2.6. Quality Assessment

Critical appraisal of the included studies was assessed using validated quality assessment tools appropriate for the study design. The Joanna Briggs Institute (JBI) critical appraisal tool [16] was used for qualitative studies; the mixed methods appraisal tool (MMAT) [17] was employed to assess mixed-method studies, and cross-sectional studies were appraised using NIH study quality assessment tool [18,19]. One reviewer completed the critical appraisal process with results verified by a second reviewer. Discrepancies were resolved by the senior author (KH). The detailed quality assessment can be found in the Supplemental Material (Table S1).

### 2.7. Data Synthesis

To summarize the literature, we narratively synthesized the study findings by mapping the results across the following key areas: (1) impact of COVID-19 on quality of life (QoL), (2) concerns related to COVID-19, (3) coping with the impact of COVID-19, and (4) recommendations for future care. Due to the methodological heterogeneity of the included literature, a meta-analysis was not considered to be an appropriate method of data synthesis.

## 3. Results

Of the 6597 articles screened, ten met the inclusion criteria. They included six quantitative cross-sectional surveys [20,21,22,23,24,25], three qualitative studies [26,27,28], and one study of mixed-method design [29]. Based on the quality assessment, six studies were of moderate quality [20,22,24,26,28,29], with three being good [21,23,25] and one study rated as poor [27]. The cross-sectional observational surveys assessed various facets of psychosocial well-being, including anxiety [20], QoL [20,21,22,23], and perceived change in attitudes and coping strategies [24,25]. Semi-structured [27,28] and structured interviews [26] were conducted to ascertain fears and concerns [26,28], changes in behavior and lifestyle [26,27], coping behaviors [27,28], and recommendations for cancer care [28] within the context of COVID-19. The included mixed-method study explored the coping strategies adopted by older adults with cancer both via a Brief-COPE questionnaire and a semi-structured interview [29]. A detailed description of the included studies is outlined in Table 1. 

The results are organized into the following themes: (1) impact of COVID-19 on quality of life (QoL), (2) concerns related to COVID-19, (3) coping with the impact of COVID-19, and (4) recommendations for future care.

**Figure 2:**
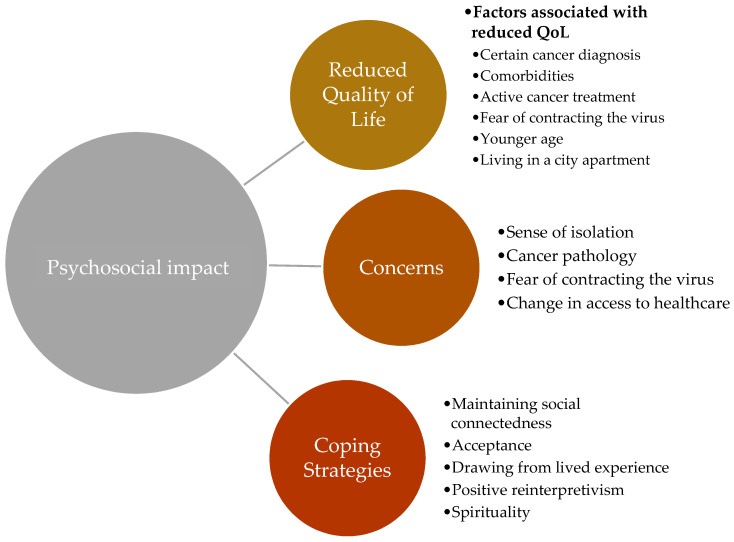
Psychosocial Impact of COVID-19 among older adults with cancer.

Figure 2 contains a visual presentation of the psychosocial impact of COVID-19 on older adults with cancer, including the factors associated with reduced QoL, major concerns amongst the population, and the adopted coping strategies. 

### 3.1. Impact of COVID-19 on Quality of Life (QoL)

The reviewed studies aimed to assess several aspects of psychosocial and emotional wellbeing, including anxiety [20], QoL, emotional functioning [20,21,22,23], and well-being and meaning in life [24,25]. Variability in psychosocial outcomes were reported across studies. 

The surveys conducted to ascertain levels of anxiety and changes in perceived psychological well-being in patients with cancer during the lockdown suggested lower levels of anxiety (GAD-7 = 3.2 ± 4.5) in areas minimally affected by COVID-19 [20]. This change met the level of minimal clinical important difference (MCID), which is estimated at 4 points on the GAD-7 total score [30]. Büssing et al. indicated a decrease in the prevalence of depressive states between the first lockdown and the second (35% vs. 31% respectively), although this failed to reach statistical significance but is considered clinically significant (estimate of 1 or more points is proposed as MCID) [24,25,31].

Variations in the QoL levels collected only during the pandemic and those comparing pre- versus during pandemic scores were observed. Both qualitative and quantitative literature conducted during the pandemic suggested a decrease in general QoL [21,26] and emotional functioning [20] during the COVID-19 lockdown and quarantine period. Interestingly, pre- versus during COVID-19 pandemic comparisons conducted by Jeppesen et al. [22] and Koining et al. [23] revealed that the global QoL and emotional functioning ascertained through EORTC QLQ-C30 were not significantly altered by the pandemic, which can be considered clinically significant (MCID estimates of 5–14 points) [32].

The included studies detail several relationships between the COVID-19 pandemic and psychosocial health. Being a woman (gender) was a risk factor for anxiety and distress induced by the pandemic (*p* < 0.03) [20,22,23]. Higher global QoL, emotional function, and wellbeing was correlated with increasing age [22], specifically, in patients aged > 60 years (*p* = 0.01) [19,24]. Those with a cancer diagnosis of brain tumors, cervical cancers, and thoracic cancers (*p*-value < 0.05) [22]; those with several comorbidities [22]; patients receiving medical cancer treatment [21], especially those receiving oral treatment [25]; those living alone (*p* < 0.05), [22]; and those residing in city apartments (*p* = 0.01) [20] fared poorer on psychosocial measures [22]. Jeppesen et al. [22] suggested that employed patients had a higher global Qol score, while Baffert et al. [20] found the same in retired individuals (*p* = 0.04). 

### 3.2. Concerns Related to COVID-19

Exploration of psychosocial wellbeing uncovered several concerns, such as loneliness, sense of frustration, concerns regarding their cancer diagnosis and treatment, fear of contracting the coronavirus, access to healthcare services, change in the pre-pandemic social structure, interruption of patients’ normal life, and a sense of uncertainty [21,22,26,27]. According to one study, before the pandemic, 18% of patients experienced some degree of isolation, with a steep increase to 67% during the lockdown [21]. 

Fears surrounding disease progression and healthcare access during the pandemic were at the fore. Patients varied in the degree to which they expressed COVID-19 related concerns across studies. Three studies concluded that more than half of their participants expressed worry about being infected by the coronavirus and having a complicated course of COVID-19 [21,22,24]. However, older adults with cancer were balancing their fear of the pandemic with their ongoing fears related to their cancer [27]; 79% of patients in one study reported being more afraid of their cancer than of COVID-19 [26]. Interestingly, those with more severe side effects and short-term cancer diagnosis focused on cancer-related concerns, whereas patients with long-term cancer diagnosis (>12 months) and those receiving ongoing treatment emphasized the threat of COVID-19 [26,27]. 

Patients had mixed feeling towards COVID-19-related social-distancing policies [27], with one study reporting 58% of participants were irritated by statements made about the dangers of a COVID-19 infection [24]. Older cancer survivors felt restricted by the pandemic due to the change in pre-pandemic social interaction, including the inability to travel or engage in formerly enjoyed social activities [18].

Concerns about change in oncology practices and access to health care were expressed by 49% of study participants [21], with 9% of patients refraining from consulting a doctor or visiting the hospital due to fear of contracting the virus [22]. Despite the fear, a majority of patients living with cancer understood the cruciality of continuing cancer treatment [27], heeding their health (80%), and keeping up with their scheduled appointments (78%) [23].

Several predictors of manifested fears and concerns towards COVID-19 were identified. Factors associated with increased distress related to COVID-19 included being a woman (gender) (55%) (*p* < 0.001), presence of comorbidities (24%), being unpartnered, having received radiotherapy or surgery for lung cancer (30%), actively receiving intravenous treatment (72%) (*p* = 0.02), or receiving oral treatment not in their residence (90%), and being younger than 60 or aged 60–70 (*p* < 0.001) [23,26,27].

### 3.3. Coping with the Impact of COVID-19

Older adults employed numerous coping strategies across the COVID-19 pandemic. Common coping strategies included maintaining social connection [24,25,27,29], redeploying previous coping strategies [27,29], and engaging with spirituality [24,25,27]. 

Despite the isolating circumstances imposed by the COVID-19 pandemic and the associated public health measures, older adults maintained social connectedness as a means of coping [25,27,29]. Büssing et al. [24,25] found participants reported more intense relationships with partners, family, and friends, along with spirituality, as a means of coping at two time points during the pandemic [24,25]. Means of maintaining social connectedness included using technology to engage with loved ones [29] and continuing to see family and friends in-person under certain perceived-safe conditions [27]. Quantitative analysis revealed that the most common strategies of older adult cancer survivors included acceptance (96.7%), self-distraction (93.3%), and taking action (93.3%) [27,29]. Older adult cancer survivors drew from their cancer and non-cancer experiences and redeployed coping strategies in the face of the current pandemic [23,29]. Hyland et al. [27] discussed how older adults with cancer used positive reinterpretation and spritituality as an important coping measure, which included focusing on the positives in life and appreciating what you have.

### 3.4. Recommendations for Future Care

Numerous studies discussed recommendations for supporting older adults with cancer during the COVID-19 pandemic. Büssing et al. [25] advocated for further spiritual care and psychotherapy as a means of supporting older adults with cancer’s resilience. Mindfulness training was recommended to clinicians with a focus on preventing ruminating on negative thoughts and instead supporting positive reinterpretation [24]. Considering the importance of maintaining social connectedness as a means of coping, Bartels et al. [21] recommended facilitating safe connection for patients and their caregivers, suggesting strategies such as peer-to-peer contact and online mental health interventions.

One of the included studies looked specifically at recommendations for supporting older adult cancer survivors across the pandemic. Haase et al. [28] conducted semi-structured, qualitative interviews with older adult cancer survivors during the pandemic. Informed by these interviews and the perspectives of older adults, recommendations for supporting older adult cancer survivors were proposed. First, healthcare providers should provide older adults with enhanced baseline information during appointments [28]. Second, healthcare providers should support the involvement of caregivers in survivorship care [28]. Third, providers should support older adults through the transition to virtual care and the further integration of technology into healthcare [28]. Finally, individuals felt people now better appreciated the value of personal protective equipment, and its use would likely be sustained beyond the pandemic [28].

## 4. Discussion

We conducted this rapid review to summarize the available literature pertaining to psychosocial well-being within the context of the COVID-19 pandemic amongst older adults with cancer. The results from the included studies were consolidated under four domains: (1) impact of COVID-19 on QoL, (2) concerns related to COVID-19, (3) coping with the impact of COVID-19, and (4) recommendations for future care. Numerous factors such as, being younger than 60 years of age [21]; being a woman [19]; having several co-morbidities [19,22]; receiving active treatment, especially oral therapies [21,25]; and living in city apartments [19,21] were associated with poorer psychosocial wellbeing and increased fears and concerns towards COVID-19. 

Interestingly, the literature reported inconsistent findings regarding QoL. Studies comparing pre-pandemic QoL scores to those collected during the pandemic show no change in the QoL and emotional functioning scores [22,23]. Baffert et al. [20] also suggested, in a region wherein the rate of infection was limited, anxiety post lockdown was low. These results were consistent with findings among patients with multiple sclerosis who reported no increase in anxiety and depression in the six months preceding the COVID-19 lockdown and during it [33]. Two possible factors could justify the results; firstly, those with a cancer diagnosis were possibly better equipped to assimilate to the COVID restriction due to their continual accommodation to a restrictive lifestyle [23]. Secondly, the increased social proximity with those within their household [23] and enhanced opportunity to allocate more time to physical activity could possibly contribute towards alleviating emotional distress [34,35]. It is noteworthy to mention that physical exercise is considered an important intervention to counteract both the physical and psychosocial impacts amongst both older adults with cancer and those without [10]. 

The surveys and qualitative studies conducted during the lockdown period alone suggest reduced QoL and emotional functioning [20,21,26]. In the included papers, depressive states were observed during both waves of the lockdown in May and September [24,25]. Similar reports of anxiety, depressive symptoms, and psychological distress during the lockdown were gathered from patients with breast cancer, ovarian cancer, thyroid cancer, and lymphoma [36,37,38,39]. The heterogeneity in cancer diagnosis, disease stage, and patient population highlights the unmet psychological needs across the cancer continuum amidst the pandemic [40,41]. Anxiety levels were higher in women [20], which is consistent with gender being a risk factor for anxiety in patients with cancer in general [42]. Similar studies conducted with healthy participants reported female gender as being significantly associated with higher levels of stress, anxiety, depression, and more negative psychological effects of COVID-19 as well [43]. Participants identifying as women expressed increased concern related to COVID-19 as they felt a responsibility for their loved ones and feared passing on the virus to them [26]. During the pandemic, heightened levels of anxiety, fears, and depression have also been observed amongst healthy community-dwelling adults as well [10]. Given the predisposing vulnerabilities of older adults with cancer, these psychosocial concerns may have more deleterious side effects [10].

Major concerns amongst older adults with cancer included an increased sense of isolation [21] and changes in access to healthcare services [21,22]. Both cancer and COVID-19 were prominent concerns of older adults with cancer; however the degree of concern varied across studies [21,22,24,26,27]. Change in cancer care, especially postponements or modifications of therapies and scheduled visits with the oncology team, were a prevalent source of distress as it meant a failure to return to normal [26,27]. Loneliness adversely affects cognitive function in older adults and confers a greater risk of poor physical and mental health [44]. Therefore, clinicians must recognize the isolation faced by older adults with cancer in order to counteract the negative impact on cognitive function [45]. Setting up mental health facilities to mitigate pandemic-induced psychological impacts of any future eventualities can be of merit [10,46].

Contrary to discourse surrounding the vulnerability of older adults, age was a reported strength in coping. Older adults above the age of 65 had better QoL scores and relatively fewer fears surrounding COVID-19 compared to their younger counterparts [20,22,23,26]. Drawing from lived experiences enabled older adults to conceptualize and cope with the current pandemic [29]. Koinig et al. [23] reported similar findings, observing that older adults with cancer were well equipped to adapt to the restrictions imposed by COVID-19, as they had previously adapted to the changes associated with their cancer diagnosis. Although the COVID-19 pandemic has caused significant disruption to older adults, so too did their cancer diagnosis. Older adults demonstrated resilience by maintaining social connectedness with family and friends either virtually [17] or through physically distant interactions [19]. Accepting the restrictions and positively reinterpreting the current social restrictions helped older adults’ express greater appreciation for their relationships, with some seeing this as an opportunity to spend more time at home [17,19]. This psychological resilience can be explained to some extent by the ‘sense of coherence theory’, which incorporates comprehensibility (ability to understand and integrate), manageability (ability to navigate and manage), and meaningfulness (sense-making) to support the better navigation of life stressors [47]. This perceived sense of coherence in the event of a new health threat is a strong predictor of health status among older adults [48,49]. This is especially beneficial as this resilience elucidates the ability of older adults to utilize both internal and external resources to facilitate successful coping with stressors, contrary to the societal discourse surrounding age-related weakness [50]. 

The present review is not without limitations. The results pooled from the included studies present a level of heterogeneity in cancer diagnosis and homogeneity in ethnicity and education within the study sample. Due to social distancing guidelines, some surveys were administered online. Hence, those with limited access to eHealth resources were at a possible disadvantage. The surveys lacked COVID-specific questionnaires to measure QoL. This provides an opportunity for the development of a tool within this realm. There is a need for future studies to have an ethnically and socioeconomically diverse sample and to employ a standardized COVID-19 specific tool to measure QoL.

## 5. Conclusions

This review, to the best of our knowledge, is the first to summarize the available evidence on the psychosocial impact of COVID-19 on older adults with cancer. The results pertaining to the change in QoL were inconsistent. Older adults were concerned by changes in their cancer care, loneliness, their disease progression, and contracting the coronavirus. Coping strategies included leaning on personal relationships, maintaining spirituality, accepting the changes associated with the pandemic, engaging in positive interpretation, and drawing on previous experience. The factors affecting the psychosocial wellbeing outlined in the current review, coupled with the suggestions for future care, can help tailor and reorganize oncology practice during the current pandemic and for any subsequent global health crises. 

## Figures and Tables

**Table 1:** Description of the study characteristic, participant details, objective, and results.

Author/Year/Country	Duration of Data Collection	DesignQuant/Qual	Sample Size (*n*)	Age (Years)	Females (%)	Cancer Diagnosis	Aim	Outcomes and Outcomes Measures	Results **
Baffert 2021 [20] France	May 2020 to the beginning of June 2020	Quant Cross-sectional survey	*n* = 189	Age range—61–70	60%	Lung, breast, and colorectal cancer	To evaluate anxiety, HRQOL during the COVID-19 pandemic, and to assess the non-psychological consequences on quality of life and satisfaction with care.	Anxiety-GAD-7 * QoL-SF-12 *	11.1% showed anxiety. Mental health deteriorated (*p* < 0.0001).
Bartels 2021 [21] Netherlands	Within two years before the start and during the COVID-19 lockdown	Quant Cross-sectional online survey	*n* = 169	Median age—68 (range 38–92)	38%	Bone metastases	To evaluate the effect of societal COVID-19 measures on changes in quality of life and emotional functioning of patients with metastatic bone disease	QoL-BPI, EORTC-C15-PAL, EORTC-BM22, and EQ5D-3L *	Decrease in general QoL (72.4 to 68.7, *p* = 0.007); increase in feeling isolated (18% before and 67% during lockdown)
Jeppesen 2021 [22] Denmark	15 May 2020 to 29 May 2020	Quant Cross-sectional cohort survey	*n* = 4571	Mean age—66	60%	Breast cancer and incurable cancer	To investigate QoL for patients with cancer, either receiving active treatment or in a follow up program during the COVID-19 pandemic with focus on emotional functioning	HRQOL—EORTC QLQ-C30 *	No clinically significant differences in global QoL and emotional function (EF) scores
Koinig 2021 [23] Austria	20 April 2020 18 June 2020	Quant Cross-sectional online survey	*n* = 240	Mean age—67	46%	Solid tumor and hematological malignancy	To study cancer patients’ perception of the COVID-19 pandemic and its impact on their everyday life during the lockdown	HRQOL—EORTC QLQ-C30 *	No clinically significant differences in physical, role, emotional, or social functioning, or of global QOL
Büssing 2021 [24] Germany	May to June 2020, (sample 1) and September to November 2020 (sample 2)	Quant Cross-sectional online survey	*n* = 292 (sample 1) *n* = 221 (sample 2)	Mean age—66.7 ± 10.8	20.1%	Prostate cancer, larynx tumours, and nasal/paranasal tumours	To analyze the change in patients’ perceptions, fear, worries, and emotional adaptation between waves 1 and 2 of the pandemic	Perceived changes- 12-item short version of the perceptions of change scale Well-being-WHO-5 * Perceived daily life affections-NAS * Meaning in life-MLQ * Indicators of spirituality-SpREUK questionnaire Awe and gratitude-GrAw-7 *	Perception of change and indicators of spirituality lower in wave 2 (*p* = 0.060).
Büssing 2020 [25] Germany	9 June to 21 June	Quant Cross-sectional online survey	*n* = 288	Mean age—66.7 ± 10.8 (range 29–92)	28%	42% prostate cancer 17% larynx tumours	To analyse whether patients with malignant tumours during the COVID-19 pandemic perceived changes of their attitudes and behaviours related to their relationships, awareness of nature and quietness, interest in spiritual issues, or feelings of worries and isolation.	Perception of Changes-12-item version of the Perceptions of Change Scale Spiritual-Religious Self-Categorization-SpREUK questionnaire Awe and Gratitude-GrAw-7 * Meaning in Life-MLQ * Well-Being Index-WHO-5 * Perception of Burden-VAS * COVID-19 Pandemic Outcomes-two single items scales Health Behaviours-Alcohol consumption	Patient wellbeing, perceived burden and perception of change was not greatly impacted by COVID-19 (*p* < 0.0001).
Catania 2020 [26] Italy	30 April 2020, to 29 May 2020	Qual Structured telephone interview	*n* = 156	Median age—68 (range 23–91)	44.2%	Lung cancer	To assess the fears associated with SARS-CoV-2 pandemic impact on lung cancer patients	Nine question qualitative survey assessing: fear of falling ill with COVID-19 compared to the fear of their disease; changes in the lives; and change in care	Quarantine period worsened the QoL of some patients (40%).
Hyland 2020 [27] USA	20 March to 8 May 2020	Qual Semi-structured telephone interview	*n* = 15	Mean age—65	60%	Lung cancer	To characterize the behavioral and psychosocial responses of people with advanced lung cancer to the COVID-19 pandemic	Interview assessing relationship of hope, goals, impact, goals, change in behavior, and psychological well-being in people with advanced stage lung cancer	Emergent themes: cancer as the primary health threat, changes in oncology practice and access to cancer care, awareness of mortality, behavioral and psychosocial responses to COVID-19, sense of loss, and positive reinterpretation/greater appreciation for life
Haase 2021 [28] Canada	June and July 2020	Qual Semi-structured telephone interviews	*n* = 30	Mean age—72.1 years (range 63–83)	57%	Breast and colorectal cancer	To report reflections on the pandemic shared by older adult cancer survivors and to understand their suggestions for suitable resources and care delivery methods	Six questions assessing concerns, coping, and changes; suggestions for future coping strategies and delivery of care	Accepted COVID restrictions, coping through positive reinterpretation
Galica 2021 [29] Canada	NR	Qual + Quant Cross-sectional survey Semi-structured telephone interviews	*n* = 30	Mean age—72.1 (range 63–83)	57%	Breast and colorectal cancer	To understand coping among older cancer survivors	Coping (quantitative data)-Brief-COPE questionnaire. (qualitative data) Telephone interview conducted to ascertain coping before and during the pandemic along with individual coping strategies	Emergent themes: (1) drawing on lived experiences, (2) redeploying coping strategies, and (3) complications of cancer survivorship in a pandemic.

* NR—not reported; GAD-7—generalized anxiety disorder screener; QoL—quality of life; HRQOL—health-related quality of life; SF-12—12-item short-form health survey; BPI—brief pain inventory; EORTC-C15-PAL and EORTC-BM22—European Organization for Research and Treatment of Cancer quality of life questionnaires; EQ5D-3L—Euro-QoL five-dimensional instrument of health-related quality of life; EORTC QLQ-C30—European Organization for Research and Treatment of Cancer Quality-of-Life-Questionnaire-Core-30; WHO-5—WHO-Five Well-being Index; NAS—numeric analogue scales; MLQ–10-item meaning in life questionnaire; GrAw-7—7-item awe/gratitude scale; VAS—visual analogue scales. ** *p*-value added where available.

## Supplementary Materials

The following are available online at https://www.mdpi.com/article/10.3390/curroncol29020053/s1, Listing S1: Search Strategy and Table S1: Quality Assessment.
