# Supplementary material for: The Psychosocial Impact of COVID-19 on Older Adults with Cancer: A Rapid Review
Source: Curr Oncol. 2022 Jan 28;29(2):589–601. doi: 10.3390/curroncol29020053 (PMC8870801; doi:10.3390/curroncol29020053)
Supplement: Supplementary file 1 [file curroncol-29-00053-s001.zip › curroncol-1560044-supplementary.pdf]

Listing S1. Search Strategy.

Search terms- MEDLINE(OVID)

Ovid MEDLINE(R) ALL <1946 to July 26, 2021>

1 exp Coronavirus/ 85818

2 exp Coronavirus Infections/ 104836

3 (coronavirus* or corona virus* or OC43 or NL63 or 229E or HKU1 or HCoV* or ncov* or covid* or sars-cov* or sarscov* or Sars-coronavirus* or Severe Acute Respiratory Syndrome Coronavirus* or "novel betacov" or "novel betacoronavirus").mp. 176694

4 ((pneumonia or covid* or coronavirus* or corona virus* or ncov* or 2019-ncov or sars*).mp. or exp pneumonia/) and Wuhan.mp. 5588

5 (2019-ncov or ncov19 or ncov-19 or 2019-novel CoV or sars-cov2 or sars-cov-2 or sarscov2 or sarscov-2 or Sars-coronavirus2 or Sars-coronavirus-2 or SARS-like coronavirus* or coronavirus-19 or covid19 or covid-19 or covid 2019 or ((novel or new or nouveau) adj2 (CoV or nCoV or covid or coronavirus* or corona virus or Pandemi*2)) or ((covid or covid19 or covid-19) and pandemic*2) or (coronavirus* and pneumonia)).mp. 161448

6 COVID-19.mp. 153723

7 1 or 2 or 3 or 4 or 5 or 6 182685

8 exp Neoplasms/ 3507441

9 (neoplasm* or cancer* or leukaemi* or leukemi* or leukeni* or tumour* or tumor* or malignan* or carcino* or lymphoma* or adenocarcinoma* or radioth* or radiat* or irradiat* or radiochemo* or chemotherap* or (bone adj marrow adj5 transplant*)).mp. [mp=title, abstract, original title, name of substance word, subject heading word, floating sub-heading word, keyword heading word, organism supplementary concept word, protocol supplementary concept word, rare disease supplementary concept word, unique identifier, synonyms] 5224091

10 8 or 9 5430660

11 exp Aged/ 3277792

12 exp Geriatrics/ 30564

13 Aging/ 236960

14 Health Services for the Aged/ 18030

15 (ageing or aging or aged or elder* or geriatric* or old age* or senior* or "older adults" or "older people" or "elderly people").mp. 5965633

16 11 or 12 or 13 or 14 or 15 5965633

17 7 and 10 and 16 2163

Table S1. Quality Assessment.

Author/year/ country/ Tool used	Question 1	Question 2	Question 3	Question 4	Question 5	Question 6	Question 7	Question 8	Question 9	Question 10	Question 11	Question 12	Question 13	Question 14	Quality Rating
Baffert 2021 France NHLBI*	Yes	Yes	Yes	Yes	No	No	Yes	NA	NA	No	Yes	NA	NA	Yes	Fair
Bartels 2021 Netherlands NHLBI*	Yes	Yes	Yes	Yes	No	Yes	Yes	NA	NA	Yes	Yes	NA	NA	No	Good
Jeppesen 2021 Denmark NHLBI*	Yes	Yes	Yes	Yes	No	No	Yes	NA	NA	No	Yes	NA	NA	Yes	Fair
Koinig 2021 Austria NHLBI*	Yes	Yes	NR	Yes	No	Yes	Yes	NA	NA	No	Yes	NA	NA	Yes	Good
Büssing 2021 Germany NHLBI*	Yes	Yes	NR	No	No	No	Yes	NA	NA	Yes	Yes	NA	NA	Yes	Fair
Büssing 2020 Germany MMAT§ Quant descriptive	Yes	Yes	Yes	Yes	Yes										Good
Catania 2020 Italy MMAT§ Quant descriptive	Yes	Yes	CD	CD	Yes										Fair
Hyland 2021 USA JBI Qual¶	Unclear	Unclear	Unclear	Unclear	Unclear	No	No	No	Yes	No					Poor
Haase 2021 Canada JBI Qual¶	Unclear	Yes	Yes	Yes	Yes	No	No	Yes	Yes	Yes					Fair
Galica 2021 Canada MMAT§	Yes	Yes	Yes	CD	1.2 Yes	1.2 Yes	1.3 Yes	1.4 Yes	1.5 Yes	4.1 Yes	4.2 Yes	4.3 CD	4.4 CD	4.5 CD	Fair

Overall appraisal- Good or Strong, Fair or Moderate, or Poor Question- Yes/ No/ CD, cannot determine/ NA, not applicable/ NR, not reported *NHLBI- National Heart, Lung and Blood Institute Quality Assessment Tool for Observational Cohort and Cross-Sectional Studies; § MMAT- Mixed Methods Appraisal Tool; ¶ JBI- Joanne Briggs Institute Critical Appraisal Tool.
